# 462. Differences in the Humoral Response to SARS-CoV-2 Infection in Children vs. Adult

**DOI:** 10.1093/ofid/ofab466.661

**Published:** 2021-12-04

**Authors:** Girlande Mentor, Olivier Drouin, Silvie Valois, Suzanne Taillefer, Christian Renaud, Fatima Kakkar

**Affiliations:** 1 Universite de Montreal, Montreal, Quebec, Canada; 2 CHU Sainte-Justine, Montreal, Quebec, Canada; 3 Centre D’infectiologie Mere Enfant, Montreal, Quebec, Canada; 4 Departement De Microbiologie, Infectiologie, Immunologie, Faculte De Medecine, Universite De Montreal, Montreal, Quebec, Canada

## Abstract

**Background:**

One of the most striking observations of the COVID-19 pandemic has been the difference in infection among children vs. adults. Overall, children with SARS-CoV-2 infection generally had milder disease compared to adults, though the cause is not clear.

The objective of this study was to compare the humoral response to infection in children vs. adults of a same family.

**Methods:**

We performed a prospective cohort study at Sainte-Justine University Health Center in Montreal, Canada from July 2020 to March 2021. Children with a positive SARS-CoV-2 PCR were recruited from the COVID-19 clinic (index case), enrollment was offered to all household members. Serum IgG against SARS-CoV-2 native S1/S2 spike proteins was measured using the Diasorin (Liaison XL) assay, 4-6 months following a positive PCR. A mean antibody threshold of 15 Arbitrary unit per ml (AU/ml) was considered seropositive, with 94.4% positive agreement to plaque reduction neutralization tests (PRNT90) at a 1:40 ratio. Antibody titer was compared between children and adults.

**Results:**

111 participants (52 adults and 59 children) were recruited from 50 separate families. Characteristic of participants and their clinical symptoms are described in Table 1. Among all participants, 76.3% children were SARS-CoV-2 seropositive vs. 51.9% of adults (p=0.007). Median antibody titer was significantly higher in children vs. adults (82.8 AU, [IQR: 18.4-130], vs 17.0 AU, [IQR: 6.8-77.8], p=0.006); findings were similar among SARS-CoV-2 PCR positive participants only. Overall, 13 participants were PCR positive but seronegative, 7 were PCR negative and seropositive, while 61 were both PCR positive and seropositive. Older participants and those with any comorbidity. Among the PCR positive group, the seropositive participants were younger (median age 31±17 vs 19±17 years, p=0.003) and more likely to have comorbidity (69% vs 29%, p=0.007).

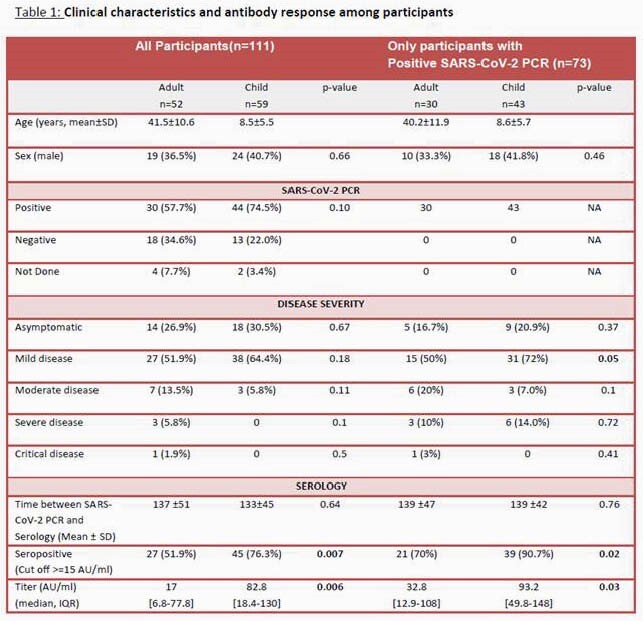

**Conclusion:**

These results suggest that children have a stronger antibody response to SARS-CoV-2 infection than adults, and that older age and presence of comorbidity are associated with a less robust humoral response. Further work on the differences in response between children and adults may help elucidate mechanisms underlying the severity of disease

**Disclosures:**

**Olivier Drouin, MDCM MsC MPH**, **Covis Pharma** (Research Grant or Support)

